# Adenosine diphosphate reduces infarct size and improves porcine heart function after myocardial infarct

**DOI:** 10.1002/PHY2.3

**Published:** 2013-05-21

**Authors:** Laurids T Bune, Jens R Larsen, Pia Thaning, Nethe E T Bune, Peter Rasmussen, Jaya B Rosenmeier

**Affiliations:** 1 The Copenhagen Muscle Research Centre, Rigshospitalet, University of CopenhagenCopenhagen, Denmark; 2 Department of Anaesthesiology-Intensive Care, Aarhus University Hospital SkejbyAarhus, Denmark; 3 Institute of Neuroscience and Pharmacology, University of CopenhagenCopenhagen, Denmark; 4 Department of Cardiology, Copenhagen University Hospital GlostrupCopenhagen, Denmark

**Keywords:** ADP, myocardial infarction, postconditioning, t-PA, UTP

## Abstract

Acute myocardial infarction continues to be a major cause of morbidity and mortality. Timely reperfusion can substantially improve outcomes and the administration of cardioprotective substances during reperfusion is therefore highly attractive. Adenosine diphosphate (ADP) and uridine-5-triphoshate (UTP) are both released during myocardial ischemia, influencing hemodynamics. Both mediate the release of tissue plasminogen activator (t-PA), which can reduce infarct size (IS). The objective of this study was to investigate whether exogenous ADP and UTP administration during reperfusion could reduce myocardial IS and whether this correlated to t-PA release or improvements in hemodynamic responses. Hemodynamic variables and t-PA were measured in 22 pigs before, during, and after 45 min of left anterior coronary artery occlusion. During reperfusion, the pigs were randomized to 240 min of intracoronary infusion of ADP, UTP, or control (no intervention). Ischemic area compared to the area at risk [IS/AAR] was measured. [IS/AAR] was 52 ± 11% in the control animals. ADP decreased [IS/AAR] by 19% (*P* < 0.05), while UTP increased [IS/AAR] by 15% (*P* < 0.05). Cardiac output (CO) increased from 3.4 to 3.5 L/min (*P* < 0.05) and mean arterial pressure (MAP) decreased from 87 to 73 mmHg in the ADP group (*P* < 0.05). t-PA concentration increased in the ADP and UTP group from 2.0 ng/mL to 2.5 and 2.4 ng/mL, respectively (*P* < 0.05) but remained unchanged in the control group. In conclusion, intracoronary ADP infusion during reperfusion reduces IS by ∼20% independently from systemic release of t-PA. ADP-induced reduction in both preload and afterload could account for the beneficial myocardial effect.

## Introduction

Acute myocardial infarction (AMI) induces rapid cell death unless blood reperfusion is quickly reestablished (Fliss and Gattinger 1996). As the amount of cell death is the primary determinant of outcome, it is a well-proven strategy to minimize the time that the myocardium is ischemic by reintroducing blood flow as soon as possible (Veinot et al. 1997). However, reperfusion per se leads to additional cell death, a process termed ischemic–reperfusion (IR) injury (Murphy and Steenbergen 2007, 2008), making the use of additional cardioprotective strategies desirable. The idea of postconditioning (POC) encompasses manipulation of the cellular events by pharmacotherapy during reperfusion, thereby reducing the amount of myocardial cell death (Zhao et al. 2003).

The cardiovascular effects of adenines (adenosine, adenosine diphosphate [ADP], and adenosine-5-triphosphate [ATP]) have been extensively studied since 1929 (Drury and Szent-Gyorgyi 1929). Adenines target specific purinergic receptors (P1, P2Y, and P2X) on the surface membrane of cardiac myocytes and vascular endothelium, causing vasodilation (Ralevic and Burnstock 1998; Burnstock 2007). Adenosine has been shown to possess cardioprotective capabilities when administered as POC. In humans, infusion of adenosine during reperfusion reduces myocardial injury although without certain improvement on clinical outcome (Ross et al. 2005).

ADP may also possess cardioprotective properties as we have recently shown that intravenous ADP infusion causes reductions in afterload and reduces myocardial oxygen demand (L. T. Bune, P. Thaning, G. R\xE5degran, Y. Hellsten, S. P. Mortensen, B. Saltin, J. B. Rosenmeier, unpubl. data). Furthermore, selective stimulation of the ADP-related endothelial P2Y_1_ receptor mediates both vasodilation and the release of tissue plasminogen activator (t-PA), a profibrinolytic enzyme released during myocardial infarction (Olivecrona et al. 2004, 2007). All these properties may be advantageous in reducing myocardial infarction.

Some purinergic P2Y-receptors also target uridine-based compounds, but only a few studies have focused on the effect in the heart.

Uridine-5-triphosphate (UTP) stimulates the P2Y_2_ and P2Y_4_ receptors. UTP has been shown to possess inotropic effects (Wihlborg et al. 2006). Furthermore, UTP infusion cause vasodilation and concomitantly inhibit sympathetic vasoconstriction (Rosenmeier et al. 2008). UTP is discharged during myocardial infarction in humans (Erlinge et al. 2005), and in rodents it has been shown to play a role in cardiac protection (Yitzhaki et al. 2006, 2007; Shainberg et al. 2009) via the P2Y_2_ receptor (Cohen et al. 2011).

In the present study, we therefore hypothesized that both ADP and UTP would exhibit cardioprotective effects in a larger animal model by reducing infarct size and improve hemodynamics following IR injury, when compared with no intervention during reperfusion. We also tested whether a given outcome was correlated with changes in t-PA release.

## Methods

This study was approved by the Danish expectorate for Animal Experimentations, and conforms with the *Guide for the Care and Use of Laboratory Animals* published by the United States National Institutes of Health (NIH Publication No. 85-23, revised 1996) regarding principles of animal care.

### Animal instrumentation

Thirty-five pigs were investigated (Danish Landrace/Yorkshire crossbreed [weight 40 kg]) and randomized into three groups: intracoronary ADP infusion (ADP), intracoronary UTP infusion (UTP), or control (CON).

The animals were premedicated with an intramuscular injection of Midazolam (Dormicum®; Roche, Basel, Switzerland) 2.5 mg/kg and anesthesia was initiated by intravenous pentobarbital (Mebumal®; DAK, Copenhagen, Denmark) 15 mg/kg and maintained with a continuous intravenous pentobarbital infusion of 15–20 mg/kg per hour. All animals received an initial bolus of 2500 IU heparin (Heparin®; Leo; Copenhagen; Denmark) and thereafter a bolus of 1500 IU per hour. After intubation, the animals were mechanically ventilated (MV 3.0–3.5 L) (S/5 Avance, Datex-Ohmeda Inc., Madison, WI) with a fresh gas flow of 6 L/min (2 L/min O_2_, and 4 L/min air). Ventilation was adjusted to ensure normal physiological blood level ranges of pH and partial pressure of carbon dioxide (PaCO_2_) throughout the experiment.

A standard ECG monitored heart rate (HR) and ST (III)-segment changes. Blood temperature (Tblood) was continuously monitored through the pulmonary catheter (CCOmbo®) and temperature was maintained between 37.3 and 38.8°C with electric warming blankets. Fluid status was ensured through infusions of 0.9% sodium chloride solution with 20 meq potassium added at a rate of 10 mL/kg per hour to replace estimated water loss and securing normohydration and s-potassium of 3.5–4.0 mmol/L).

Catheters were introduced in the left jugular vein and in the left carotid artery for blood samples, fluid infusions, and mean arterial pressure (MAP) recordings. The right jugular vein was used for direct pressure measurement (S/5 Avance) via a pulmonary artery catheter (CCOmbo®, Edwards Lifesciences LLC, Irvine, MN) for blood sampling and to measure cardiac output (CO), pulmonary arterial pressure (PAP), and mixed venous oxygen saturation (SVO_2_) and were connected to a Baxter Vigilance cardiac output monitor (Edwards Life Sciences, Irvine, CA).

### Experimental protocol

**Figure 1:**
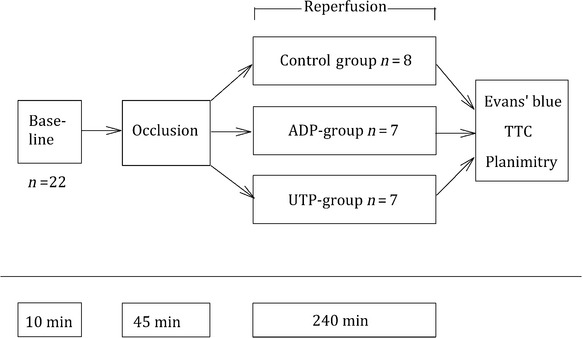
Experimental protocol. Baseline values were obtained after a resting period of at least 15 min to ensure stable values and 10 min before occlusion. Occlusion was maintained for 45 min and reperfusion for 240 min. During the occlusion period, the pigs were divided into three groups. One control group, one group receiving intracoronary adenosine diphosphate (ADP) during the reperfusion period, and one group receiving intracoronary uridine triphosphate (UTP) during the reperfusion period.

Throughout the experiments, continuous measurements of HR, MAP, PAP, SVO_2_, CO, PaCO_2_, Tblood, and ST (III)-segment changes were recorded every 10 sec (Fig. 1).

After a 15-min resting period, baseline values were recorded. A standard left coronary angiography was performed with a size 4 JL-type catheter placed via a sheath in the right carotid artery. Under contrast-enhanced fluoroscopy, a 9-mm length balloon-tipped percutaneous coronary intervention (PCI) catheter was guided into the left anterior descending artery (LAD) and positioned distal to the second diagonal terminal branch. The LAD was then occluded by inflating the 2.5-mm diameter PCI catheter for 45 min at 7 bars. Total occlusion was verified by contrast-fluoroscopy, and PCI placement was recorded digitally before and after inflation. After deflating the PCI catheter, a reperfusion period of 240 min followed. During this period, the animals allocated to active treatment, would have either UTP or ADP infused directly into the LAD through the JL-catheter.

### Drug infusions and concentrations

Drug concentration was chosen based on previous studies on the relative vasoactive potency of ADP and UTP (Rosenmeier et al. 2008; Bune et al. 2010) where UTP>>>ADP in the peripheral circulations of humans. The infusion rate was kept at 2 mL/min which has been shown not to affect coronary blood flow(Olivecrona et al. 2004). ADP was infused at 10 μmol/min and UTP was infused at 1 μmol/min (both Sigma, St. Louis, MO). Infusion was initiated as the reperfusion period began and continued throughout the rest of the experiment. Arrhythmias were treated immediately with either direct-current counter shock or chest compressions. Cardiac shock was treated with external cooling and Trendelenburg position. No additional medicine was given.

### Infarct area calculation

**Figure 2:**
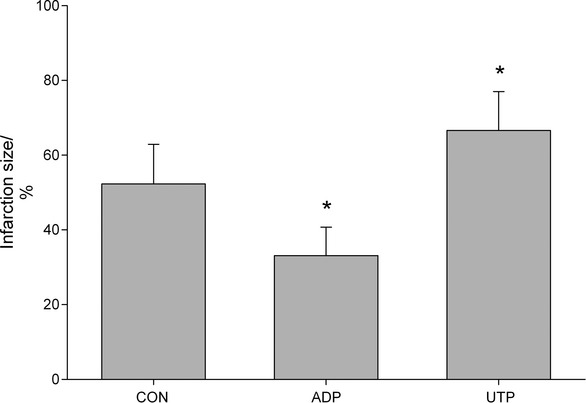
Myocardial infarct size [IS/AAR] in all three groups after 45 min and LAD occlusion/240 min reperfusion. Data are presented as mean ± SD. Groups: Controls (*n* = 8), UTP infusion (*n* = 7), ADP infusion (*n* = 7) showing that ADP infusion reduce [IS/AAR] (*P* = 0.003) and UTP infusion increases [IS/AAR] infarct areas (*P* = 0.028). Control group: [IS/AAR] = 52 ± 11%. ADP group: [IS/AAR] = 33 ± 8%. UTP group: [IS/AAR] = 67 ± 10%.

The size of the ischemic risk area (area at risk, AAR) and infarct size (IS) were measured postmortem following in vivo contrast stain with Evans' blue dye and ex vivo histochemical staining with 2-,3-,5-triphenyl-tetrazolium chloride (TTC)(Fishbein et al. 1981) (Fig. 2).

Briefly; the heart was exposed through a median sternotomy after the reperfusion period. The LAD artery was externally ligated at the position of the PCI-balloon occlusion, and Evans' blue dye (8% w/v) was injected directly into the left atrium of the beating heart. The AAR was demarked as tissue colored area, and healthy myocardium is demarked dark blue. The heart was then excised, frozen for 45 min at −80°C and then transected into 6–8 slices along the long axis plane. Thereafter, the slices were scanned with a high-resolution digital photo-scanner for digital image recordings. Following this, the slices were incubated in TTC 1% w/v in phosphate buffer solution (pH 7.40) for 10 min at 37°C. TTC stains viable tissue red and leaves infarct tissue white. The slices were then rescanned and weighed. Computerized planimetry, with a user-dedicated macro application of web-based freeware (Image J© 1.35n, National Institute of Health, Bethesda, MD) were used to standardize measurements of the size of AAR and IS in a blinded fashion. The weight-standardized areas were used to calculate the ratio [IS/AAR].

### Blood samples

Blood samples were collected from the aorta and the right atrium at baseline (0 min), and at 40 min, 46 min, and 105 min.

Blood samples were analyzed for hemoglobin concentration, O_2_ saturation, PaCO_2_, and PaO_2_ (1.5 mL; ABL605 and OSM 3 Hemoximeter, Radiometer, Copenhagen, Denmark).

Plasma concentrations of t-PA (ng/mL) were determined by using commercial ELISA kits (TintElize t-PA, Biopool AB, Sweden). All samples were performed in duplicate.

### Statistical analysis

[IS/AAR] was tested using one-way analysis of variance (ANOVA). Pairwise differences were identified using Tukey's post hoc procedure. For the hemodynamic parameters and t-PA two-way repeated measures analysis of covariance (ANCOVA) was used to identify changes, with time as the within-subjects factor and drug dose as the between-subjects factor. Values defined as the last 10 min before the reperfusion and treatment start were used as covariates and following a significant *F*-test, Dunnett's post hoc procedure was used for determination of pairwise differences. SAS (proc mixed, ver. 9.1, SAS Institute Inc., Cary, NC) was used for the analysis. *P* < 0.05 was considered statistically significant and data are presented as mean with 95% confidence intervals or where appropriate with SD.

## Results

Of 35 randomized pigs, 22 completed the experiments. Three animals died during instrumentation, six animals died during the ischemic period, and four animals died during reperfusion (two control animals and two animals receiving UTP). Of the four pigs that died during reperfusion, three died from acute intractable heart failure (two controls, one UTP) and one from intractable ventricular fibrillation (one UTP).

For all the hemodynamic variables no significant differences were observed between the control and treatment groups at baseline or during ischemia (data not shown). Hemodynamic parameters remained stable during ischemia (Table 1). Furthermore, during the entire experiment no difference in Tblood or PaCO_2_ between groups was observed.

**Table 1:** Baseline values and values during occlusion before interventions

	*N*	Baseline	Occlusion
HR (bpm)	22	70.8 ± 17.7	78.1 ± 20.1
MAP (mmHg)	22	91.9 ± 15.2	85.7 ± 14.6
CO (L/min)	20	3.6 ± 1.3	3.4 ± 1.0
PAP (mmHg)	21	23.5 ± 8.8	27.2 ± 10.3
SVO_2_ (mL/min)	22	68.7 ± 12.0	65.3 ± 12.2

No difference between groups was observed. HR, heart rate; MAP, mean arterial pressure; CO, cardiac output; PAP, pulmonary artery pressure; SVO_2_, systemic venous oxygen content.

### Drug infusion influence on myocardial infarct size after coronary reperfusion

Myocardial infarct size was 52 ± 11% in the control group (Fig. 2). Infusion of ADP resulted in a significant reduction in myocardial [IS/AAR] when compared to the control group. In the ADP group [IS/AAR] was reduced by 19 (14–25)% (*P* = 0.003). In contrast, the UTP infusion increased [IS/AAR] by 15 (7–22)% when compared to the control animals (*P* = 0.028).

### Hemodynamic effects of drug infusion during reperfusion

In the control animals, HR increased from 78 (68–88) beats per minute (bpm) during ischemia to 102 (91–112) bpm during reperfusion (*P* < 0.0001). With ADP and UTP treatment, HR increased to 100 bpm (96–104, *P* < 0.0001) and 99 bpm (95–103, *P* < 0.0001), respectively, and not different from the control animals.

**Figure 3:**
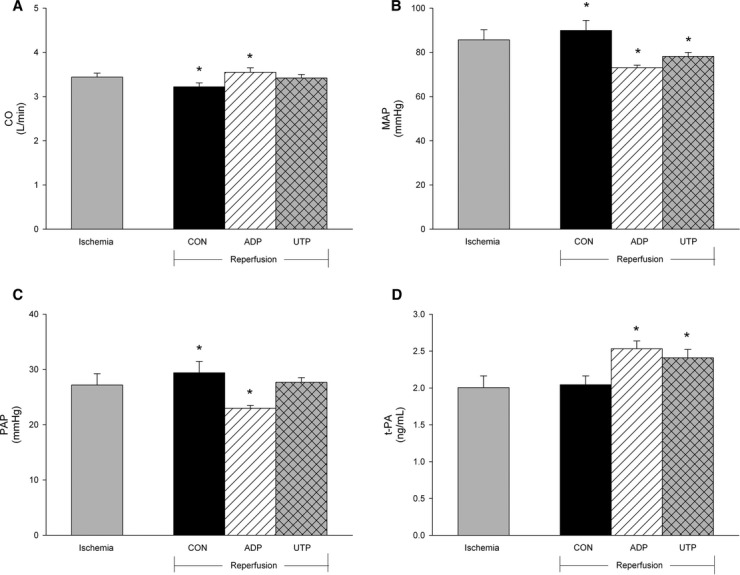
Changes in (A) cardiac output (CO), (B) mean arterial pressure (MAP), (C) pulmonary arterial pressure (PAP), and (D) tissue plasminogen factor (t-PA) during ischemia and reperfusion. *Significant difference from ischemia *P* < 0.05 and all data are presented as mean ± SD.

Cardiac output was 3.4 (3.2–3.7) L/min during ischemia and decreased to 3.2 (3.0–3.5) L/min during reperfusion in control animals (*P* < 0.0001; Fig. 3A). During reperfusion, the ADP group increased CO to 3.5 (3.3–3.8; *P* < 0.0099) L/min when compared to ischemia. For the UTP group, there was no change (*P* = 0.68).

In the control animals, SVO_2_ decreased from 65 (63–67)% during ischemia to 62 (60–65)% during reperfusion (*P* < 0.0001). Conversely, in the ADP group, SVO_2_ increased to 67% (65–69; *P* = 0.0007) during reperfusion as did the UTP group (to 67 [65–69]%; *P* = 0.007). Both groups were significantly different from the controls, *P* = 0.0002 and *P* < 0.0001, respectively.

MAP increased from 87 (74–97) mmHg to 90 (78–102) mmHg in the control animals (*P* < 0.0001; Fig. 3B). In contrast to this, ADP lowered MAP to 73 (70–75) during reperfusion (*P* < 0.0001). Also, UTP decreased MAP to 78 (74–83) mmHg during cardiac reperfusion (*P* < 0.0001). Both treatment groups were different from the control group (*P* = 0.0011 and *P* = 0.0476, respectively).

During ischemia, mean pulmonary artery pressure (PAP) was 27 (22–33) mmHg in controls and reperfusion increased PAP to 29 mmHg (24–35; *P* < 0.0001) (Fig. 3C). In contrast, ADP infusion resulted in a decrease in PAP to 23 mmHg (22–24; *P* < 0.0001), which was different from the controls (*P* = 0.0078). No changes were observed in the UTP group in comparison to controls.

### t-PA

t-PA was 2.0 (1.7–2.3) ng/mL at the end of ischemia in the control animals. No change was observed during reperfusion (*P* = 0.0040).

In the ADP and UTP treatment group, t-PA increased to 2.5 ng/mL (2.3–2.7; *P* = 0.0023) and 2.4 ng/mL (2.2–2.6; *P* = 0.025), respectively, during cardiac reperfusion (Fig. 3D).

## Discussion

The aim of this study was to evaluate the infarct size and cardioprotective effects of coronary ADP and UTP administration during the reperfusion period after myocardial infarction in comparison to no intervention. There are three novel findings in the present study: (1) infusion of ADP during reperfusion reduced myocardial infarct size; (2) in contrast to previous studies in rodents, infusion of UTP increased myocardial infarct size; and (3) t-PA had no influence on myocardial infarct size.

This study demonstrates that intracoronary ADP infusion can lead to less myocardial necrosis, reflected in a reduced infarct size by 19% in comparison to control and by 34% in comparison to UTP.

The preservation of myocardial function by ADP could be the result of a combined reduction in both afterload and preload during the infusion whereas UTP only reduced afterload. HR did not differ between intervention groups, thus leading to a relative improvement in stroke volume in the ADP group. The difference in CO between ADP and controls therefore indicated a better functioning left ventricle (LV). The preserved LV function by ADP administration was further testified by an increase in SVO_2_.

The potential mechanisms of ADP-induced cardioprotection are several. ADP could act on a cellular level as a precursor to ATP-generation via endothelial ATP synthesis and thereby help to restore energy balance/supplies, essential for myocyte survival. It is unlikely, however, that ADP would enter the endothelial cells in concentrations needed to generate the ATP needed for energy restoration. Another possibility is that ADP is dephosphorylated to adenosine, which has known cardioprotective effects in experimental models (Sommerschild and Kirkeboen 2000; Morrison et al. 2007). However, recent studies using different endogenous adenine ligands have demonstrated that lower concentrations of ADP are needed to elicit a cardiovascular response in comparison to adenosine (Rosenmeier et al. 2008; Bune et al. 2010). It is therefore plausible that ADP acts directly on endothelial or myocardial P2Y_1_ or P2Y_12_ receptors, possibly leading to nitric oxide (NO) generation (Buvinic et al. 2002). NO has been shown to possess cardioprotective capabilities when used as treatment (Yang et al. 2004). Previous studies in a canine model demonstrated that low-dose intravenous NO infusion reduce left ventricular (LV) preload, improve regional perfusion, and reduce infarct size. However, this only occurs when NO is carefully titrated to decrease mean blood pressure by 10% but not below 80 mmHg, during early stages of acute myocardial infarction (Jugdutt 1994). Whether ADP also needs the same extend of careful titration cannot be determined from the present study.

Although ADP is well known for its thrombogenic role in platelet aggregation (Vilen et al. 1985), this was clearly not a problem in the current study, as no reocclusion occurred. Whether this was due to stimulation of P2Y_1_ receptors, which mediate both t-PA release and vasodilation (Olivecrona et al. 2004, 2007), is unknown, but it is in concordance with a precious study, demonstrating that continuous ADP infusion leads to a reduced coagulative capacitance (Bune et al. 2010).

A previously demonstrated cardioprotective role of UTP in rodents could not be confirmed in this study. The administration of UTP during reperfusion in P2Y_2_ receptor knock-out mice reduced infarct size, proposing a protective effect of UTP through inhibition of cytosolic and mitochondrial Ca^2^^+^-uptake (Yitzhaki et al. 2006; Shainberg et al. 2009; Cohen et al. 2011). Rodents, however, have very high resting heart rates at 250–300 bpm, and the calcium homeostasis required for such high heart frequency is very different from human and pig cardiomyocytes. It is possible that rodents exaggerate the extent of this particular cardioprotective pathway and the inconsistency could be due to differences in the animal models.

It could be argued, that the detrimental effect of UTP in the current investigation is the result of a lower infusion rate than in the ADP group. However, in a pilot study (*N* = 2), we observed that using intracoronary infusion rates at ∼1 μg UTP/min resulted in severe tachycardia which is not beneficial in an acute infarction setting. Also, as UTP previously was demonstrated to be more potent than ADP in the human peripheral circulation in equimolar concentrations(Rosenmeier et al. 2008), we selected a lower infusion rate to prevent the risk of coronary hyperperfusion, and aimed at inducing similar HR-changes and MAP-reductions for the two drugs. We, therefore, consider it unlikely that the 34% difference in infarct size between UTP and ADP caused by differences in coronary blood flow but may reflect distinctly different receptor effects and/or secondary metabolic effects between the two compounds. A possible explanation could be that UTP is far more dependent upon endothelial-derived hyperpolarization factor (EDHF) release to cause vasodilatation (Vanhoutte et al. 2009), and it does not cause release of NO to the same extent as ADP. Another mechanism may be due to the extent of reperfusion injury as UTP may be a still be better vasodilator in the coronary circulation in comparison to ADP although the infusion rate was lower.

In the present study, we did not find that endogenously released t-PA to be related to the development of myocardial infarction. This is in contrast to what has been previously suggested (van der Pals et al. 2010). It is worth noticing that the therapeutic concentration of exogenous t-PA has been reported to be 1 mg/kg (Zivin et al. 1985) and therefore is much higher than the concentration measured in the present study.

### Limitations

The present study was performed on 4-month-old pigs, and careful evaluation must be applied due to the difference in anatomy, physiology, and biochemistry between a porcine experimental model and humans with atherosclerotic vasculature and endothelial dysfunction. As we did not use any selective P2 blockers, it is not possible to determine whether the cardioprotective effect seen by ADP is due to a direct effect on the one of the specific ADP-related receptor itself or due to activation of secondary pathways. As with all physiologic mechanisms, redundancy may very likely also occur and two to triple blockade studies are very challenging in animal models. Whether modulation of secondary receptor pathways causes the cardioprotective effect needs to be further investigated by using selective inhibitors.

Also, measurements of the concentrations of ADP, UTP as well as their degradation products in the LAD, the coronary sinus, the aorta, and in jugular vein would have given additional insight of which metabolites that may be responsible of the effects observed in the present study.

## Conclusion

Intracoronary ADP administration during reperfusion significantly reduces infarct size and improves cardiac function. The effect of ADP seems related to a favorable hemodynamic profile and action directly on the myocardium as it is not related to t-PA release. It could therefore be speculated that selective P2Y_12_ antagonist combined with P2Y_1_ agonist can improve ischemic POC and thereby have clinical consequences for the treatment of myocardial infarction.
